# Damage-Associated Molecular Patterns and Trained Immunity of Monocyte–Macrophage Populations in Osteoarthritis: Potential Molecular Links and Therapeutic Opportunities

**DOI:** 10.3390/cells15191756

**Published:** 2026-09-26

**Authors:** Jian Zhang, Bingbing Chen, Yundong Xu, Heguo Yan, Niqin Xiao, Jing Xie, Zhaohu Xie, Zhaofu Li

**Affiliations:** 1 The First Clinical Medical College, Yunnan University of Chinese Medicine, Kunming 650500, China; 17860503891@163.com (J.Z.);; 2 College of Basic Medical Sciences, Yunnan University of Chinese Medicine, Kunming 650500, China

**Keywords:** osteoarthritis, trained immunity, damage-associated molecular patterns, pattern recognition receptors, monocyte–macrophage populations, epigenetic reprogramming, metabolic reprogramming

## Abstract

**Highlights:**

**What are the main findings?**

- Direct human evidence supports an HMGB1-associated trained phenotype in circulating monocytes from knee OA, although broader OA-specific validation remains limited.
- DAMP sensing may converge with epigenetic and immunometabolic reprogramming to sustain altered monocyte–macrophage responsiveness in OA.

**What are the implications of the main findings?**

- Evidence stratification is essential for distinguishing demonstrated OA-specific relationships from mechanisms extrapolated from other inflammatory conditions.
- The interface between DAMP signaling and trained immunity provides testable targets, but causal and phenotype-specific validation is required before clinical translation.

**Abstract:**

Osteoarthritis (OA) is now widely discussed as a whole-joint immunometabolic disease involving cartilage, synovium, and subchondral bone, rather than as cartilage degeneration driven only by mechanical stress. Tissue injury, cell death, mitochondrial dysfunction, and extracellular matrix degradation can lead to the persistent release of damage-associated molecular patterns (DAMPs), which activate innate immune responses through pattern recognition receptors (PRRs). A key unresolved issue is why inflammation can persist or recur when there is no continuous external stimulus. Trained immunity provides an emerging framework for approaching this problem, with initial direct evidence now available in circulating monocytes from knee OA but broader validation still lacking. Following an initial stimulus and a subsequent resting interval, monocytes and macrophages may retain epigenetic and metabolic alterations that modify their responses to later homologous or heterologous challenges. This review summarizes the sources of DAMPs and their recognition mechanisms in the OA microenvironment, with particular attention to their potential links with trained immunity in monocyte–macrophage populations. We discuss PRR-mediated changes in chromatin accessibility, histone modifications, DNA methylation, and non-coding RNA regulation, as well as immunometabolic events such as enhanced glycolysis, tricarboxylic acid cycle remodeling, mitochondrial dysfunction, and accumulation of key metabolites. We also examine how monocyte–macrophage populations with persistent hyperresponsiveness after stimulus withdrawal and restimulation may contribute to persistent synovitis, cartilage matrix degradation, and abnormal subchondral bone remodeling. Finally, we summarize therapeutic opportunities potentially relevant to the proposed links between DAMP signaling and trained immunity. Recent evidence that high-mobility group box 1 (HMGB1) primes a trained phenotype in circulating monocytes provides initial direct support for DAMP-associated trained immunity in knee OA. However, its generalizability across independent cohorts, other DAMP classes, myeloid-cell compartments, disease stages, and OA phenotypes remains to be established.

## 1. Introduction

Osteoarthritis (OA) is characterized by progressive cartilage degeneration, abnormal subchondral bone remodeling, synovial inflammation, and disruption of the local joint microenvironment. With a high prevalence, substantial disability burden, and prolonged disease course, OA represents an increasing global health challenge [1,2]. Traditionally, OA has been considered primarily a degenerative disorder caused by mechanical stress-induced cartilage wear. However, accumulating evidence indicates that OA is not merely a passive degenerative process but a chronic disease involving complex immune responses. Synovial inflammation, immune cell infiltration, and sustained production of inflammatory mediators are increasingly recognized as important contributors to structural damage and persistent pain [3]. Recent single-cell sequencing and transcriptomic studies have further demonstrated that OA-associated inflammation is not characterized by nonspecific immune activation, but rather by remodeling of myeloid cell populations, enhanced macrophage inflammatory programs, and altered intercellular communication networks [4]. Consistently, single-cell atlases of human knee joints have revealed an increased proportion of inflammatory pain-associated macrophages and a reduction in regenerative stem/progenitor cell populations within OA tissues, suggesting that immune dysregulation is deeply involved in joint degeneration [5].

During the development of persistent inflammation in OA, damage-associated molecular patterns (DAMPs) have been recognized as important upstream signals linking tissue injury to immune activation. DAMPs mainly originate from damaged cells, cell death processes, and extracellular matrix (ECM) degradation products. Under sterile conditions, these endogenous danger signals can be continuously recognized by pattern recognition receptors (PRRs), thereby amplifying innate immune responses and maintaining inflammatory imbalance within the OA joint microenvironment [6]. However, persistent DAMP exposure alone cannot fully explain why myeloid immune cells, particularly monocyte–macrophage populations, remain hyperresponsive upon subsequent stimulation. The concept of trained immunity provides a potential framework for understanding this phenomenon. Trained immunity refers to a long-lasting functional reprogramming state of innate immune cells following an initial stimulus, which enables enhanced responses upon secondary challenges [7]. Importantly, trained immunity should be distinguished from persistent immune activation caused by the continued presence of an initiating stimulus. Persistent activation depends on ongoing stimulation and generally subsides after the stimulus is removed, whereas trained immunity persists during a stimulus-free interval and is revealed by an altered response to subsequent restimulation. Therefore, the demonstration of trained immunity generally requires a training–rest–restimulation design and evidence that the altered functional state persists after withdrawal of the initial stimulus. This process is mainly regulated by coordinated epigenetic and metabolic reprogramming. Epigenetic alterations, including changes in chromatin accessibility, histone modifications, DNA methylation, and non-coding RNA regulation, reshape the transcriptional responsiveness and activation thresholds of inflammatory genes. Meanwhile, metabolic remodeling, characterized by enhanced glycolysis, tricarboxylic acid (TCA) cycle remodeling, mitochondrial alterations, and accumulation of key metabolites, contributes to the maintenance of trained states and regulation of inflammatory outputs [8,9].

Importantly, direct OA-specific evidence has recently begun to emerge. A recent study identified a trained phenotype in circulating monocytes from patients with knee OA and demonstrated that high-mobility group box 1 (HMGB1) contributes to this functional reprogramming [10]. This finding provides initial direct support for the proposed relationship between DAMP exposure and trained immunity in OA. Nevertheless, because the evidence currently derives from a limited number of studies, independent replication and extension to other DAMPs, myeloid-cell populations, joint compartments, and OA phenotypes remain necessary.

Although DAMP biology, synovial macrophage heterogeneity, immunometabolism, epigenetic regulation, and trained immunity have each been reviewed in OA or other chronic inflammatory diseases, these topics have generally been discussed as separate domains. Previous OA-centered reviews have primarily focused on the inflammatory effects and therapeutic targeting of individual DAMPs, whereas macrophage-centered reviews have emphasized cellular heterogeneity, polarization, and intercellular communication. In parallel, trained-immunity reviews have largely derived their mechanistic frameworks from infection, vaccination, atherosclerosis, and other inflammatory disorders. The distinctive contribution of the present review is therefore not a separate summary of these established fields, but an evidence-stratified integration of the sequence linking DAMP exposure, PRR-dependent sensing, monocyte–macrophage reprogramming, altered responsiveness to secondary challenge, and the persistence of inflammation in OA. This framework also distinguishes experimentally demonstrated OA-specific relationships from supportive observations and mechanistic extrapolations.

Against this background, this review critically evaluates the potential molecular links between DAMP exposure and trained immunity of monocyte–macrophage populations and examines whether these relationships may help explain the persistence and recurrence of inflammation in OA. We summarize the major sources of DAMPs and their recognition pathways in the OA microenvironment, with a focus on the monocyte–macrophage lineage because these cells combine DAMP sensing, functional plasticity, inflammatory amplification, and persistent functional reprogramming. We further discuss how epigenetic and metabolic reprogramming may jointly establish inflammatory memory and how this process could contribute to sustained synovitis, cartilage matrix degradation, and abnormal subchondral bone remodeling. Finally, we examine therapeutic opportunities arising from the potential links between DAMP signaling and trained immunity and discuss their translational relevance. Throughout the article, we distinguish direct OA evidence from mechanisms inferred from broader trained-immunity research, because trained immunity in OA is currently best regarded as a testable model rather than a fully established disease mechanism.

## 2. Literature Search Strategy

This narrative review was informed by literature searches conducted in PubMed and the Web of Science Core Collection from database inception to 31 July 2026. The search strategy combined terms related to osteoarthritis, trained immunity, monocyte–macrophage populations, DAMPs, PRRs, and epigenetic and metabolic reprogramming. The principal search terms included “osteoarthritis,” “knee osteoarthritis,” “trained immunity,” “innate immune memory,” “monocyte,” “macrophage,” “damage-associated molecular pattern,” “DAMP,” “HMGB1,” “S100A8/A9,” “extracellular matrix fragment,” “pattern recognition receptor,” “TLR,” “RAGE,” “NLRP3,” “epigenetic reprogramming,” “immunometabolism,” and “metabolic reprogramming.” These terms were used in different combinations to identify relevant studies. The reference lists of eligible articles and recent reviews were also screened to identify additional publications.

Evidence derived from human OA monocytes, macrophages, synovial tissue, or synovial fluid was prioritized. OA animal and in vitro studies were included to evaluate cell-specific and tissue-level mechanisms potentially linking DAMP signaling to trained immunity. Because several molecular features of trained immunity have not been directly investigated in OA, selected non-OA studies using established training–rest–restimulation paradigms were incorporated to define mechanistic plausibility. Such studies were explicitly identified as extrapolative evidence and were not considered direct evidence of trained immunity in OA. Particular attention was given to publications addressing the persistence of functional changes after stimulus withdrawal, responses to secondary challenge, and associated epigenetic or metabolic reprogramming.

To improve transparency, the evidence was classified into four categories: (A) direct human OA evidence, including functional or molecular evidence obtained from OA-derived monocytes or macrophages; (B) OA animal or in vitro evidence that supports individual components of the proposed pathway but does not establish the complete trained-immunity process; (C) trained-immunity evidence from non-OA human or experimental models; and (D) mechanistic inference or hypothesis. Epigenetic or metabolic abnormalities observed exclusively in OA chondrocytes or other non-myeloid cells were treated as contextual OA evidence rather than direct evidence of monocyte–macrophage trained immunity.

## 3. Sources, Release, and Sustained Accumulation of DAMPs in the Osteoarthritic Joint Microenvironment

DAMPs in the OA joint microenvironment arise from multiple pathological processes, including ECM fragmentation, cellular injury or death, crystal deposition, and mitochondrial dysfunction (Figure 1). Repetitive mechanical overload and incompletely resolved cartilage, meniscal, or ligament injury can continuously generate matrix fragments and cellular debris, particularly in post-traumatic OA. Their repeated release and inefficient clearance sustain low-grade innate immune activation through PRRs and may create conditions permissive for myeloid-cell training. Experimentally, intermittent DAMP pulses generated by repeated mechanical loading should be distinguished from uninterrupted DAMP exposure: temporally separated stimulation permits assessment of persistence during stimulus-free intervals, whereas continuous exposure primarily evaluates sustained activation, adaptation, or tolerance. However, evidence that a DAMP activates a PRR or induces inflammatory mediator production does not demonstrate that it produces a persistent memory state after stimulus withdrawal. Among the DAMPs discussed below, HMGB1 currently has the strongest direct evidence for inducing a trained monocyte phenotype in knee OA, whereas most ECM fragments, S100 proteins, crystals, and mitochondrial components have been shown primarily to activate or prime inflammatory pathways.

### 3.1. ECM Degradation-Derived DAMPs

ECM degradation represents one of the earliest and most persistent sources of DAMPs during OA progression. Beyond reflecting structural deterioration, ECM breakdown generates bioactive matrix fragments known as matrikines, which possess immunomodulatory properties. Proteolytic fragments derived from ECM components, including collagen, aggrecan (ACAN), fibronectin (FN), and hyaluronan (HA), can serve as endogenous signals that continuously promote inflammatory and catabolic responses [11]. Among these molecules, the III13-14 domain of FN can activate TLR4 signaling in joint tissues and synergize with interleukin-1 (IL-1) and tumor necrosis factor (TNF) to amplify local inflammatory responses [12]. Similarly, ACAN-derived fragments can induce OA-associated pain phenotypes through TLR2, indicating that ECM fragments contribute not only to inflammatory persistence but also to disease-related symptoms [13].

The immunological activity of HA is also highly dependent on its molecular size. Low-molecular-weight HA (LMW-HA) preferentially activates the Toll-like receptor 4 (TLR4)/nuclear factor kappa B (NF-κB) axis and promotes inflammatory responses, whereas high-molecular-weight HA (HMW-HA) generally exhibits tissue-protective and immunoregulatory effects. However, fragmented HA does not consistently display DAMP-like pro-inflammatory activity across all arthritis models, suggesting that its biological effects are influenced by molecular size, cell type, and inflammatory context [14,15,16]. Therefore, ECM degradation products in OA should not be considered merely structural debris but rather biologically active sources that continuously deliver danger signals.

### 3.2. Cellular Injury, Necrosis, and Alarmin-DAMP Release

Cellular injury within the OA joint is accompanied by several forms of regulated cell death that differ in their capacity to release DAMPs. Mechanical stress, oxidative stress, and mitochondrial dysfunction can induce chondrocyte apoptosis, pyroptosis, necroptosis, and ferroptosis [17,18]. Classical apoptosis is generally non-lytic and may remain immunologically silent when apoptotic cells are efficiently removed through efferocytosis; substantial DAMP release becomes more likely when clearance is defective and apoptotic cells undergo secondary necrosis. In contrast, pyroptosis and necroptosis are inherently membrane-disruptive, whereas ferroptosis is associated with lipid peroxidation and progressive loss of membrane integrity. Membrane disruption or defective efferocytosis facilitates the extracellular release of intracellular molecules, including HMGB1 and S100 calcium-binding proteins A8/A9 (S100A8/A9), which can function as alarmins and promote sterile inflammation [17,18,19]. Thus, the magnitude and composition of DAMP exposure depend on both the mode of cell death and the efficiency of cellular clearance.

Unlike the transient inflammatory response associated with acute injury, continuous cell death and DAMP release in OA may establish a positive feedback loop that maintains prolonged immune activation within the joint. Clinical studies have shown that HMGB1, S100A8/A9, and receptor for advanced glycation end products (RAGE) levels are associated with OA activity, suggesting that these molecules represent components of a persistent pathological signaling axis rather than merely biomarkers of tissue damage [20,21]. Thus, the significance of alarmins extends beyond indicating the occurrence of tissue injury; they actively participate in maintaining the post-injury inflammatory state. Among these alarmins, HMGB1 currently has the strongest direct OA-specific evidence for inducing a trained phenotype in circulating monocytes [10], whereas comparable evidence for S100A8/A9 remains unavailable.

### 3.3. Crystal Deposition and Mitochondria-Derived DAMPs

In addition to soluble DAMPs, particulate danger signals contribute to the inflammatory environment of OA. Basic calcium phosphate (BCP) crystals can alter the metabolic state of monocyte-derived macrophages and promote a pro-inflammatory phenotype [22,23], while calcium pyrophosphate dihydrate (CPPD) crystals may coexist with OA and amplify episodic joint inflammation [24]. Monosodium urate (MSU) crystals should also be considered, particularly in patients with hyperuricemia or coexisting gout, because OA synovium may facilitate MSU deposition, cellular uptake, and subsequent inflammatory responses [25]. However, evidence that BCP, CPPD, or MSU crystals induce bona fide trained immunity in OA-derived myeloid cells is currently lacking.

Mitochondrial injury represents another important source of endogenous danger signals. Mitochondrial damage can lead to the release of mitochondrial DNA (mtDNA) into the extracellular environment. Studies have shown that mtDNA levels in synovial fluid are elevated following joint injury and correlate with cartilage damage severity, suggesting that mitochondrial disruption and extracellular nucleic acid release may serve as persistent stimuli contributing to inflammatory maintenance [26]. Moreover, mtDNA-associated genetic backgrounds have been linked to OA susceptibility, indicating that mitochondrial dysfunction may not merely accompany disease progression but may also contribute to disease predisposition [27]. Taken together, crystal deposition and mitochondria-derived DAMPs constitute a persistent and difficult-to-clear danger-signal reservoir within the OA joint and may sustain innate immune activation through continuous PRR engagement.

### 3.4. PRR-Mediated Sensing of DAMPs and Inflammatory Signal Integration

The transition from DAMP exposure to sustained inflammatory amplification requires receptor recognition and downstream signal integration. TLR4 is one of the major PRRs involved in DAMP sensing in OA. Activation of TLR4 induces transcriptional programs involving NF-κB, activator protein-1 (AP-1), and interferon regulatory factor 3 (IRF3), which are closely associated with the maintenance of low-grade inflammation within the joint microenvironment [28]. In addition to TLR4 signaling, the HMGB1/RAGE axis represents another important danger-sensing pathway in OA. RAGE not only mediates the recognition of HMGB1 and related ligands but also participates in chondrocyte regulation, inflammatory amplification, and tissue remodeling [29]. Although OA is generally characterized by sterile inflammation, endogenous DAMPs and microbial pathogen-associated molecular patterns (PAMPs) may converge on shared PRRs. Both systemic and synovial lipopolysaccharide (LPS) burdens have been associated with knee OA severity and local inflammation [30], while increased circulating LPS has also been observed in obesity-related OA [31]. Thus, low-grade PAMP exposure may cooperate with locally released DAMPs by increasing basal PRR signaling or lowering the threshold for subsequent myeloid-cell activation. However, these associations do not establish that combined DAMP–PAMP exposure induces trained immunity in OA-derived monocytes or macrophages.

Unlike cell-surface or endosomal danger-sensing receptors such as TLRs and RAGE, NOD-, LRR- and pyrin domain-containing protein 3 (NLRP3) does not generally function as a classical receptor that directly binds a specific extracellular DAMP. Instead, NLRP3 acts as a cytosolic inflammasome sensor and signaling platform that responds to cellular disturbances generated downstream of diverse danger signals, including potassium efflux, lysosomal damage, mitochondrial dysfunction, and reactive oxygen species (ROS). Subsequent assembly of the NLRP3 inflammasome activates caspase-1, promotes IL-1β maturation, and contributes to inflammatory amplification and pain in OA [32]. Therefore, DAMPs in OA should not be viewed as isolated products of tissue injury. Instead, they participate in a broader inflammatory network involving cell-surface or endosomal danger-signal recognition, downstream NLRP3 inflammasome activation, and inflammatory amplification. These processes may create conditions permissive for trained immune reprogramming but do not, by themselves, demonstrate the establishment of trained immunity in monocyte–macrophage populations. However, ECM fragments, alarmins, crystals, and mtDNA should not all be considered established inducers of trained immunity merely because they trigger acute PRR activation or sustain inflammatory signaling. At present, direct OA-specific evidence supports HMGB1 as a trained-immunity-inducing DAMP in circulating monocytes [10], whereas the other DAMPs discussed above should be regarded as candidate training stimuli until validated using training–rest–restimulation designs. To complement the pathway-level overview shown in Figure 1, the cellular context, experimental models, and evidence level of the principal DAMP–PRR relationships discussed in this review are summarized in Table 1.

## 4. Biological Basis of Trained Immunity and Its Relevance to OA

Trained immunity is not simply a residual consequence of persistent inflammation. Rather, it is a persistent functional state acquired following an initial training stimulus, which, in sterile inflammatory diseases, may include DAMPs. Its defining feature is the reprogramming of cellular response thresholds and functional outputs, allowing subsequent challenges to elicit altered or amplified responses. In the context of OA, where chronic low-grade sterile inflammation persists within the joint microenvironment, this altered state may contribute to recurrent inflammatory activation and disease progression.

### 4.1. Core Characteristics and Biological Functions of Trained Immunity

Trained immunity is a form of innate immune memory characterized by enhanced responsiveness following an initial stimulus through persistent functional reprogramming. This process is primarily mediated by epigenetic and metabolic alterations that reshape transcriptional profiles and subsequent response patterns [33,34]. Unlike adaptive immune memory, trained immunity does not depend on antigen-specific recognition or clonal expansion. It also differs from endotoxin tolerance, which reduces cellular responsiveness following repeated stimulation [35,36]. Importantly, trained immunity is context-dependent and does not invariably promote inflammation. For example, alveolar macrophages can acquire a pro-resolution phenotype through Krüppel-like factor 4 (KLF4)–MER proto-oncogene tyrosine kinase (MERTK)-mediated efferocytosis [37]. Thus, trained immunity may amplify chronic sterile inflammation or, under different conditions, support tissue repair and inflammatory resolution.

Trained immunity can be induced by PAMPs or DAMPs. Cytokines and metabolic factors may further amplify or modify these programs but should be distinguished from the primary danger signals that initiate them. These stimuli activate PRR-dependent signaling and may induce persistent changes in cellular responsiveness [38,39]. In chronic sterile inflammatory conditions, DAMPs released from ongoing tissue damage may provide repeated or low-intensity training inputs [40,41]. However, the induction and durability of trained immunity depend on the identity, intensity, duration, and frequency of the stimulus, as well as the metabolic–epigenetic plasticity of the responding cells. Metabolic alterations provide substrates and cofactors for epigenetic regulation, while epigenetic modifications may stabilize the resulting functional state [42]. Trained immunity is not restricted to mature monocytes and macrophages. Evidence from non-OA models indicates that hematopoietic stem and progenitor cells (HSPCs) can retain epigenetic and metabolic alterations and transmit modified response programs to their myeloid progeny [43]. This progenitor-level training may contribute to the long-term persistence of innate immune memory, although it has not been directly demonstrated in OA. To distinguish trained immunity from persistent activation, transient priming, and immune tolerance, the operational criteria are summarized in Table 2.

Accordingly, DAMP-associated trained immunity in OA should be tested using a defined training–rest–restimulation paradigm. OA-derived circulating monocytes or synovial macrophages should be exposed to a candidate DAMP, followed by stimulus washout and a defined resting interval before homologous or heterologous restimulation. Enhanced TNF, IL-1β, or IL-6 production should be accompanied, where possible, by persistent chromatin or metabolic changes assessed using assay for transposase-accessible chromatin using sequencing (ATAC-seq), histone H3 lysine 4 trimethylation (H3K4me3) and histone H3 lysine 27 acetylation (H3K27ac) profiling, metabolic-flux analysis, or metabolomics. Parallel continuous-stimulation and tolerance conditions are required to determine whether the observed phenotype represents memory rather than ongoing activation or reduced responsiveness.

### 4.2. Relevance of Trained Immunity to OA

OA provides a pathological environment compatible with the induction of trained immunity, including persistent DAMP exposure, chronic low-grade inflammation, and continuous remodeling of myeloid cell functions [44]. Aging, obesity, and metabolic dysfunction may modify this environment by increasing basal inflammatory tone, altering myeloid-cell metabolism, and impairing inflammatory resolution. Inflammaging may further lower the threshold at which repeated DAMP or PAMP exposure produces persistent changes in innate immune responsiveness. Obesity-associated factors are particularly relevant because circulating LPS is increased in obesity-related OA [31], and leptin can induce long-term hyperresponsiveness in human monocytes after stimulus withdrawal and restimulation [45]. These factors should therefore be regarded as modifiers of the probability, magnitude, and durability of innate immune training rather than as evidence of trained immunity by themselves.

Within this context, trained immunity offers a potential framework for understanding inflammatory persistence and recurrent disease progression in OA. Traditional models have largely attributed OA-associated inflammation to local tissue injury and inflammatory mediator release; however, this view does not fully explain why inflammatory responses persist or readily recur in certain patients. The trained-immunity concept suggests that initial injury events may alter the functional state of local immune cells, rendering them more sensitive to subsequent stimuli and establishing a sustained inflammatory predisposition [46]. At the cellular level, monocytes and macrophages exhibit substantial functional plasticity and represent key effector populations involved in trained immunity. Within the OA joint microenvironment, these cells participate in inflammatory signaling, tissue remodeling, and immune regulation, making them potential intermediates linking tissue injury, immune-state alteration, and disease progression. Of note, current evidence does not demonstrate that OA is entirely driven by trained immunity. Rather, OA displays several features compatible with trained immune responses, including persistent stimuli, appropriate responding cell populations, and a chronic relapsing trajectory [47]. Therefore, trained immunity may serve as a complementary framework for explaining the persistence and recurrence of OA-associated inflammation, while integrating with existing concepts of degeneration and inflammation.

## 5. Monocyte–Macrophage Populations Potentially Involved in Trained Immunity in OA

For DAMPs to be converted into persistent inflammatory responses in OA, they must be sensed and processed by myeloid cells capable of coordinating sequential processes including recruitment, recognition, amplification, and memory formation. Monocyte–macrophage populations represent a central cellular component of this proposed process. Following recruitment into the joint, circulating monocytes differentiate into synovial macrophages with context-dependent functional states and are further shaped by local DAMP exposure and intercellular signals. These changes may reflect activation, priming, or polarization and should be classified as trained immunity only when persistent altered responsiveness is demonstrated after stimulus withdrawal and restimulation. Thus, monocyte–macrophage populations serve as critical cellular mediators linking DAMP signaling to sustained immune reprogramming.

### 5.1. Recruitment and Functional Priming of Circulating Monocytes

The recruitment of circulating monocytes into OA joints is not a random process but is guided by specific chemokine axes. Among these, the C-C motif chemokine ligand 2/C-C motif chemokine receptor 2 (CCL2/CCR2) axis promotes monocyte recruitment, inflammatory propagation, and cartilage destruction, indicating that it represents a key pathway controlling monocyte infiltration during OA progression [48]. In addition, the C-C motif chemokine ligand 3/C-C motif chemokine receptor 1 (CCL3/CCR1) axis further facilitates the recruitment of CD14^+^CD16^−^ circulating monocytes into inflamed synovial tissues and contributes to disease progression [49]. Of note, monocytes in OA are not maintained in a completely quiescent state. Human studies have shown that peripheral monocytes from patients with OA exhibit features of functional priming, including increased expression of CD16, CCR2, and human leukocyte antigen-DR (HLA-DR), together with enhanced production of TNF and IL-1β. These alterations are associated with inflammatory status, body mass index (BMI), and pain severity [50]. These findings indicate systemic activation or priming but do not establish trained immunity because persistence after removal from the OA inflammatory environment and altered responsiveness to subsequent stimulation were not assessed. More direct evidence has recently extended these observations from inflammatory priming to trained immunity. Single-cell transcriptomic analysis identified a distinct circulating monocyte subtype in patients with knee OA, characterized by increased expression of interleukin 1 alpha (IL1A), interleukin 1 receptor type 1 (IL1R1), TNF, several C-C and C-X-C motif chemokine ligands, and G0/G1 switch 2 (G0S2) [10]. These cells exhibited features of trained immunity and showed C-X-C motif chemokine receptor 4 (CXCR4)-dependent recruitment to the synovium, where they aggravated progressive synovial inflammation. These findings suggest that at least one component of OA-associated trained immunity may be established in circulating monocytes before their migration into the joint. However, whether this phenotype persists after monocytes differentiate into synovial macrophages or is shared by other circulating monocyte subsets remains unknown. At the synovial fluid level, CD14^+^ monocytes and soluble CD14 (sCD14) are also associated with OA progression. sCD14 can promote fibroblast-like synoviocytes (FLSs) to produce IL-6, IL-8, and matrix metalloproteinase-3 (MMP-3), while recurrent joint effusions contain increased proportions of CD14^+^ monocytes, suggesting that monocytes undergo functional alterations both before and after entering the joint environment [51]. Taken together, circulating monocytes currently represent the best-supported cellular compartment of trained immunity in OA. Recent evidence demonstrates that HMGB1 induces a trained phenotype in these cells and promotes their CXCR4-dependent migration into the synovium, thereby providing a potential peripheral-to-joint route for transferring trained inflammatory responses [10].

### 5.2. Functional Heterogeneity of Synovial Resident and Infiltrating Macrophages

Synovial macrophages are not a uniform population but rather represent a dynamic system composed of resident and infiltrating subsets with distinct origins and functional characteristics. Under homeostatic conditions, resident macrophages located in the synovial lining contribute to joint integrity by forming a protective barrier and removing cellular debris. These cells exhibit CX3C chemokine receptor 1 (CX3CR1)-associated residency characteristics and are linked to homeostatic programs involving MERTK, triggering receptor expressed on myeloid cells 2 (TREM2), and lymphatic vessel endothelial hyaluronan receptor 1 (LYVE1) [52,53]. During OA progression, this protective barrier is disrupted, accompanied by macrophage expansion and functional diversification. While some macrophage populations retain tissue repair and remodeling properties, others shift toward proliferative and inflammatory amplification programs [54]. Transcriptomic analyses of OA synovium have revealed enrichment of macrophage-associated inflammatory programs involving CCL3/CCR1, TNF, and macrophage migration inhibitory factor (MIF) pathways [4]. Moreover, synovial fluid-derived monocyte–macrophage lineage cells can be further classified into multiple subsets, including CD14^+^CD16^−^ monocyte-like cells and CD14^+^CD16^−^ macrophage-like cells, indicating that infiltrating cells do not represent a fixed terminal state but rather a continuously remodeled cellular spectrum within the synovial cavity [55]. In this context, resident macrophages are primarily associated with barrier maintenance, debris clearance, and inflammatory restraint, whereas infiltrating macrophages more closely resemble CCR2/CCR1-driven inflammatory effectors.

However, although the heterogeneity and plasticity of these populations identify the synovial compartment as a plausible local substrate for trained immunity, persistent trained states have not yet been demonstrated in either resident or recruited synovial macrophages using a training–rest–restimulation design. Long-lived, locally renewing resident macrophages represent a more plausible substrate for retaining inflammatory memory within the joint, whereas recruited monocyte-derived macrophages may transfer systemically acquired trained programs into the synovium.

Because circulating monocytes are relatively short-lived, long-term inflammatory memory in chronic OA is unlikely to reside exclusively in the same peripheral monocyte population. Its persistence may instead involve repeated recruitment of trained monocytes, self-maintaining resident synovial macrophages, reprogramming of bone marrow HSPCs, and sustained systemic metabolic or inflammatory signals. The relative contribution of these cellular substrates remains unresolved in OA.

### 5.3. Interactions Between Monocyte–Macrophage Populations and Joint Cellular Networks During Inflammatory Amplification

The central role of monocyte–macrophage populations in OA is also attributed to their position within the broader network of cellular interactions in the joint. OA synovial fluid can establish a pro-inflammatory niche that promotes a pro-inflammatory macrophage state, indicating that the local fluid environment itself is sufficient to reshape myeloid cell functions [56]. In animal OA models and human OA samples, studies of CD14^+^ macrophages have shown that CCR2-derived recruited macrophages can activate FLS through the oncostatin M/oncostatin M receptor (OSM/OSMR) signaling network, thereby establishing an inflammatory amplification loop between macrophages and synovial stromal cells [57]. At the clinical level, interventions targeting synovial macrophages and their functional states can alleviate OA-associated pain, suggesting that this cellular network contributes not only to structural damage but also to pain persistence [58]. In addition, OA synovial fluid biomarker profiles are consistent with inflammatory signatures associated with activated macrophages and neutrophils and correlate with structural damage and disease severity, indicating that monocyte–macrophage activation is integrated into the OA disease phenotype [59]. Therefore, monocyte–macrophage populations should not be considered isolated effector cells. Instead, they represent important regulatory nodes connecting DAMP stimulation, intercellular communication, and the maintenance of chronic inflammation in OA.

## 6. Potential Molecular Links Between DAMP Signaling and Trained Immunity in OA

The potential relationship between DAMP signaling and trained immunity cannot be explained simply by sustained activation of individual inflammatory pathways. Instead, the proposed framework involves the integration of initial danger-signal sensing, persistent functional alterations, epigenetic remodeling, and metabolic reprogramming (Figure 2). Endogenous danger signals are first detected through PRR-dependent pathways and may alter cellular response thresholds. These functional changes may subsequently be stabilized through chromatin remodeling and metabolic network reconfiguration. As a result, monocyte–macrophage populations may exhibit lower activation thresholds and enhanced inflammatory responses upon secondary stimulation. In OA, these processes may contribute to the transition from transient sterile inflammation to persistent or recurrent inflammatory states. However, most OA-specific evidence currently supports individual components of this framework rather than a complete causal sequence.

### 6.1. Initial Stimulation and PRR Activation

A critical step in DAMP-induced trained immunity is whether the initial stimulus is sufficient to drive cells into a primed state through specific PRRs and their adaptor proteins. In OA, this process mainly occurs during the continuous sensing of endogenous danger signals by chondrocytes, synovial cells, and monocyte–macrophage populations. Increasing evidence suggests that DAMPs released from injured tissues are not merely passive by-products of damage but can actively reshape receptor expression profiles and cellular response thresholds. For example, FN fragments can upregulate TLR2 expression in human articular chondrocytes and induce matrix metalloproteinase (MMP) expression through a myeloid differentiation primary response 88 (MyD88)-dependent pathway, suggesting that initial stimulation itself can enhance cellular sensitivity to subsequent DAMP exposure [60,61]. The outcome of initial stimulation is determined not only by the presence of DAMPs but also by the specific PRRs and adaptor molecules engaged by these signals. The catabolic and hypertrophic effects induced by LMW-HA and HMGB1 in mouse chondrocytes depend on the TLR2/TLR4-MyD88 signaling axis, and deletion of MyD88 markedly reduces DAMP-induced catabolic responses [62]. These findings indicate that PRR activation does not merely initiate immediate inflammatory responses but also participates in establishing future cellular response thresholds during the initial stimulation phase. Consistent with this concept, upregulation of the cartilage matrix protein lumican in OA enhances TLR4-dependent cartilage degradation and macrophage pro-inflammatory polarization, further suggesting that certain ECM-derived DAMPs can initiate inflammatory and metabolic programs that may be relevant to myeloid-cell training, although direct memory formation has not been demonstrated [63].

In addition to protein and matrix-derived fragments, nucleic acid-derived DAMPs also contribute to the initiation of trained responses. Double-stranded RNA (dsRNA) released from damaged chondrocytes promotes cartilage degeneration through the TLR3/interleukin-33 (IL-33) axis, while TLR3 deficiency alleviates degenerative changes in joints, indicating that endosomal nucleic acid sensing represents another important danger-signal entry pathway in OA [64]. In addition, the biological activity of DAMPs is not determined solely by their abundance but is also regulated by molecular conformation and receptor-associated cofactors. For instance, the pro-inflammatory activity of HMGB1 depends on its redox state; disulfide HMGB1 requires interaction with myeloid differentiation protein 2 (MD-2) to efficiently activate TLR4 signaling [65]. Beyond its established role in acute inflammatory signaling, HMGB1 has recently been directly linked to trained immunity in circulating monocytes from knee OA. HMGB1 exposure increased chromatin accessibility at multiple inflammatory and chemokine-associated loci and enhanced inflammatory responses following subsequent HMGB1 challenge [10]. In the human ex vivo arm of that study, circulating monocytes from patients with knee OA, whose antecedent in vivo training exposure could not be experimentally timed, were isolated, maintained for a 2-day resting period, and then restimulated with recombinant HMGB1 (40 ng/mL); IL-18 (1 ng/mL) and CCL4 (50 ng/mL) were used as comparison stimuli. Only HMGB1 elicited a stronger inflammatory response in OA-derived than in healthy-donor monocytes [10]. However, the duration of the antecedent HMGB1 exposure and the restimulation incubation was not explicitly reported. Thus, the study functionally supports a rest–restimulation response but does not reproduce a fully controlled in vitro training–washout–restimulation protocol. Mechanistically, MyD88 promoted the nuclear recruitment of interferon regulatory factor 1 (IRF1), while tripartite motif-containing protein 28 (TRIM28)-mediated small ubiquitin-like modifier (SUMO) conjugation enhanced the stability of IRF1 within chromatin and increased the transcriptional accessibility of pro-inflammatory genes. The HMGB1–MyD88–IRF1 pathway therefore currently provides the most direct OA-specific connection between a defined DAMP, persistent chromatin remodeling, and an enhanced response to restimulation. Whether the same molecular program operates in recruited monocyte-derived macrophages or long-lived resident synovial macrophages remains unknown. Of note, initial stimulation does not always result in unidirectional amplification. Recent studies in OA have shown that sialoglycans can regulate the interaction between sialic acid-binding immunoglobulin-like lectin 5 (Siglec-5) and TLR4, thereby modulating the responsiveness of TLR4 to OA-associated ligands [66]. This supports the possibility that the initiation of trained immunity by DAMPs is determined by the balance between activating and inhibitory receptor signals. Taken together, the effects of initial DAMP exposure depend on ligand properties, PRR engagement, adaptor proteins, and the balance between activating and inhibitory signals. These factors may alter subsequent cellular response thresholds and create conditions permissive for epigenetic and metabolic reprogramming.

### 6.2. Epigenetic Reprogramming

Epigenetic reprogramming represents a fundamental basis through which trained immunity persists beyond the resting phase and enables rapid reactivation upon secondary stimulation. Its essence does not lie in the continuous presence of inflammatory mediators, but rather in the ability of initial stimuli to remodel specific genomic loci into a more accessible and responsive state, allowing subsequent danger signals to induce stronger transcriptional responses at lower activation thresholds. However, OA-specific epigenetic evidence is currently derived predominantly from cartilage and chondrocytes and should therefore be interpreted as evidence of an altered joint epigenetic environment rather than direct evidence of trained immunity in monocyte–macrophage populations.

#### 6.2.1. Chromatin Accessibility and Transcriptional Remodeling

The enhanced responsiveness observed during secondary stimulation is first reflected by alterations in chromatin accessibility and transcriptional availability. Studies have shown that β-glucan can partially reverse the LPS-induced tolerant state of monocytes, restoring active epigenetic features at previously silenced inflammatory and phagocytosis-related loci and recovering their responsiveness to subsequent stimulation [67]. These findings indicate that trained immunity does not simply prolong the initial inflammatory response; rather, it reconfigures specific genomic regions that were previously inaccessible or partially restricted into a state that can be readily activated. Further studies have demonstrated that transient inflammatory stimulation can establish a myeloid-biased open chromatin landscape in hematopoietic stem cells through a CCAAT/enhancer-binding protein β (C/EBPβ)-dependent mechanism, thereby generating enhanced innate immune responses upon secondary infection [68]. Thus, chromatin remodeling associated with trained immunity is not limited to mature monocytes and macrophages but may also involve upstream hematopoietic regulatory processes. In the context of OA, this suggests that persistent DAMP exposure may not require complete reconstruction of inflammatory programs after each stimulation event. Once relevant genomic loci enter a primed chromatin state, local immune cells may become more susceptible to subsequent activation. ATAC-seq analyses have revealed extensive aberrant enhancer accessibility in human OA cartilage, with many altered regions associated with genes involved in ossification, mesenchymal stem cell (MSC) differentiation, and inflammatory regulation [69]. These findings demonstrate a persistently altered chromatin landscape in OA cartilage but do not establish comparable remodeling in myeloid cells. Whether repeated DAMP exposure induces persistent chromatin-accessibility changes in OA monocytes or synovial macrophages remains to be determined.

#### 6.2.2. Histone Modifications

If chromatin accessibility determines whether specific genomic regions can be rapidly activated, histone modifications determine whether these regions can maintain a heightened responsive state over extended periods. Representative active marks associated with trained immunity include H3K4me3 and H3K27ac. In primary human monocytes, transient stimulation with oxidized low-density lipoprotein (oxLDL) enhances TNF and IL-6 production upon secondary stimulation and is accompanied by increased H3K4me3 enrichment at inflammatory gene promoters, indicating that trained states can be stably recorded at key genomic loci [70]. In addition, fumarate accumulation resulting from glutaminolysis can inhibit lysine demethylase 5 (KDM5) activity, thereby maintaining H3K4me3 levels and stabilizing trained immune programs [71]. These findings demonstrate that H3K4me3 and H3K27ac are closely associated with rapid inflammatory gene activation during secondary stimulation. Abnormal histone modifications also contribute to the disruption of OA tissue homeostasis. In OA chondrocytes, increased expression of the histone H3K27 demethylase ubiquitously transcribed tetratricopeptide repeat on chromosome X (UTX) is associated with reduced SRY-box transcription factor 9 (SOX9) expression and impaired ECM synthesis, whereas UTX deficiency preserves cartilage integrity and attenuates cartilage damage, synovitis, and osteophyte formation in experimental OA [72]. Because this study was conducted in chondrocytes and did not assess persistence after stimulus withdrawal or responses to secondary challenge, it demonstrates cell-specific epigenetic regulation in OA rather than trained immunity in monocyte–macrophage populations. Separately, expression of the histone H3 lysine 36 methyltransferase nuclear receptor-binding SET domain protein 1 (NSD1) is reduced in aging and OA cartilage, and its deficiency is associated with impaired chondrocyte differentiation and disrupted matrix homeostasis [73]. Together, these studies establish histone-modifying enzymes as regulators of chondrocyte dysfunction and cartilage homeostasis in OA. However, they do not demonstrate persistent histone reprogramming or trained immunity in OA monocyte–macrophage populations.

#### 6.2.3. DNA Methylation

Compared with chromatin accessibility and histone modifications, DNA methylation represents a relatively slower regulatory layer in trained immunity, influencing whether functional memory can persist over longer time scales and maintain stable response biases. Studies have shown that following neonatal Bacillus Calmette–Guérin (BCG) vaccination, circulating monocytes retain DNA methylation signatures for more than 12 months, with these modified regions enriched in antiviral and inflammatory response genes. This supports the possibility that long-term maintenance of trained immunity may involve stable DNA methylation remodeling rather than relying solely on transient histone modifications [74]. O6-methylguanine-DNA methyltransferase (MGMT) is primarily a DNA-repair enzyme that removes alkylation-induced O6-methylguanine lesions and should not be regarded as conventional DNA methylation machinery. Nevertheless, MGMT deficiency attenuates β-glucan-induced trained immunity in bone marrow-derived macrophages, as indicated by reduced pro-inflammatory cytokine production following secondary stimulation and alterations in pathways involving the farnesoid X receptor (FXR), AMP-activated protein kinase (AMPK), mechanistic target of rapamycin (mTOR), and hypoxia-inducible factor 1 alpha (HIF-1α) [75]. These findings implicate MGMT-dependent DNA repair and metabolic regulation in trained immunity but do not demonstrate that MGMT establishes innate immune memory through canonical DNA methylation. Genome-wide DNA methylation analyses of OA cartilage have revealed systematic differences between OA and healthy cartilage, with aberrant methylation sites enriched in genes associated with inflammation, matrix degradation, and transcriptional regulation [76]. Peripheral blood DNA methylation-based models can also predict radiographic progression and pain development in OA [77]. However, methylation signatures in cartilage or peripheral blood indicate tissue-specific or systemic epigenetic alterations and do not establish trained immunity in myeloid cells without cell-resolved analyses and functional restimulation experiments.

#### 6.2.4. Regulation by Non-Coding RNAs

Non-coding RNAs provide an additional layer of fine-tuned regulation for epigenetic memory. Long non-coding RNAs (lncRNAs) can influence the reactivation efficiency of inflammatory genes by regulating three-dimensional chromatin organization and transcriptional complex recruitment. A subset of these molecules, termed immune gene-priming lncRNAs (IPLs), has been shown to participate in the maintenance of H3K4me3-associated trained programs [78]. In OA, increased expression of lncRNA H19 alleviates inflammatory responses and apoptosis in chondrocytes, suggesting that local lncRNA networks may regulate inflammatory thresholds within the joint microenvironment [79]. MicroRNAs (miRNAs) also contribute to the connection between metabolic alterations and epigenetic memory. For example, miR-9-5p regulates β-glucan-induced trained immunity by suppressing isocitrate dehydrogenase 3α (IDH3α), and its depletion reduces IL-1β, IL-6, and TNF production by monocytes following secondary stimulation [80]. In the OA microenvironment, mechanically overloaded macrophages can transfer miR-350-3p to chondrocytes through extracellular vesicles. This miRNA downregulates NSD1 and reduces histone H3 lysine 36 methylation (H3K36me1/2), ultimately promoting cartilage catabolism and cellular senescence [81]. This finding demonstrates miRNA-mediated epigenetic communication between macrophages and chondrocytes but does not directly establish a trained state in either cell population. For circular RNAs (circRNAs), direct evidence linking them to classical trained immunity remains limited. However, recent studies have demonstrated that extracellular circRNAs can be rapidly and efficiently taken up by macrophages and subsequently participate in antigen presentation or translation processes, suggesting their potential role as stable carriers of danger signals and long-term regulatory inputs [82]. In OA, hsa_circ_0005567 is downregulated in synovial tissues, and its overexpression promotes a reparative and inflammation-resolving macrophage state through the miR-492/suppressor of cytokine signaling 2 (SOCS2) axis, thereby reducing chondrocyte apoptosis [83]. Thus, under conditions of sustained DAMP exposure in OA, non-coding RNAs may function primarily as regulators of intercellular communication and cellular response thresholds.

### 6.3. Metabolic Reprogramming

Metabolic reprogramming provides both the energetic and signaling basis for the induction and maintenance of trained immunity. Trained monocytes and macrophages redistribute glucose, glutamine, and mitochondrial substrate utilization, allowing metabolic intermediates to support ATP production, epigenetic regulation, and inflammatory transcription. In OA, sustained DAMP exposure may create a metabolic environment permissive for the development or maintenance of hyperresponsive myeloid-cell states. However, the available evidence is strongly cell-type-dependent. Most direct evidence for trained-immunity-associated metabolic reprogramming derives from monocytes and macrophages in non-OA models, whereas many OA-specific findings originate from chondrocytes. The latter should therefore be regarded as contextual evidence of OA-associated metabolic dysregulation rather than direct evidence of trained immunity in OA myeloid cells.

#### 6.3.1. Enhanced Glycolysis and TCA Cycle Remodeling

One of the most consistent metabolic features of trained immunity is enhanced glycolysis. β-Glucan induces aerobic glycolysis in human monocytes through the protein kinase B (AKT)/mTOR/HIF-1α axis, whereas inhibition of mTOR or HIF-1α impairs the trained response [84]. Non-microbial stimuli can induce related changes. In primary human monocytes, oxLDL enhances both glycolysis and oxygen consumption, while glycolytic inhibition or metformin attenuates inflammatory responses following secondary stimulation [85]. These findings indicate that trained immunity involves coordinated remodeling of glycolytic and mitochondrial carbon flux rather than an exclusive shift from oxidative phosphorylation (OXPHOS) to glycolysis. Metabolic alterations have also been observed in OA-associated cells. IL-1β or TNF enhances glycolysis and reduces mitochondrial respiration in OA chondrocytes [86]. Patient-matched analyses further demonstrate reduced entry of glucose-derived carbon into the TCA cycle, impaired ATP production, decreased mitochondrial membrane potential, and abnormal mitochondrial morphology in OA chondrocytes [87]. Glycolytic inhibition can reduce the expression of matrix-degrading genes under inflammatory conditions. These findings establish OA-associated metabolic dysregulation but do not, by themselves, demonstrate trained immunity in monocytes or macrophages. In particular, glycolytic switching should be interpreted as a potential component of trained immunity only when it persists after withdrawal of the primary stimulus and is associated with an altered response to secondary challenge.

#### 6.3.2. Accumulation of Key Metabolites

The persistence of trained immunity is partly supported by the signaling and epigenetic functions of specific metabolites. In β-glucan-trained monocytes, enhanced glutaminolysis leads to fumarate accumulation, which contributes to the maintenance of training-associated epigenetic changes [71]. Succinate has a more immediate role in inflammatory amplification. In LPS-stimulated macrophages, succinate stabilizes HIF-1α and promotes IL-1β expression [88]. Although this evidence was obtained outside OA, hypoxia, acidosis, and persistent danger signaling within OA synovium may favor activation of the succinate/HIF-1α pathway. Its involvement in trained immunity of OA macrophages nevertheless remains inferential. Lactate may connect metabolic reprogramming more directly with persistent functional changes. It can support the TCA cycle in trained monocytes and enhance trained responses through histone lactylation [89]. In OA, hyperglycemia-associated lactate accumulation induces CD11b lactylation in synovial macrophages, impairs efferocytosis, and aggravates synovitis [90]. Thus, fumarate and lactate have clearer links to established trained-immunity or OA macrophage pathways, whereas the role of succinate in OA myeloid-cell training remains hypothetical.

#### 6.3.3. Mitochondrial Function, Reactive Oxygen Species, and the mTOR/HIF-1α Axis

Mitochondrial function also influences the establishment and maintenance of trained immunity. In oxLDL-trained macrophages, enhanced OXPHOS and persistent changes in mitochondrial morphology and function contribute to the secondary inflammatory response, whereas inhibition of mitochondrial respiration weakens this response [91]. Thus, trained immunity involves coordinated glycolytic and mitochondrial remodeling rather than complete suppression of OXPHOS.

ROS may connect mitochondrial dysfunction with inflammatory amplification. In OA chondrocytes, lactate dehydrogenase A (LDHA)-mediated glycolysis increases ROS production, whereas its inhibition reduces oxidative stress and cartilage damage [92]. This finding supports metabolic–oxidative coupling in OA but does not directly demonstrate trained immunity in OA macrophages. Whether persistent DAMP exposure produces durable mitochondrial and ROS-dependent reprogramming in OA-derived myeloid cells remains to be established.

The mTOR/HIF-1α axis represents a central regulatory node integrating these metabolic changes. mTOR promotes HIF-1α stabilization and induces glycolysis-related gene expression, while HIF-1α converts metabolic alterations into functional outputs by regulating metabolic enzymes, lactate production, and inflammatory gene transcription [84,88]. In OA, this axis exhibits clear cell type- and stage-dependent effects. In synovial macrophages, it may support the maintenance of pro-inflammatory metabolic programs, whereas in chondrocytes, moderate HIF-1α stabilization can alleviate apoptosis and cartilage degeneration by enhancing mitophagy and reducing oxidative stress [93,94]. Therefore, metabolic reprogramming in OA cannot be simply described as enhanced glycolysis accompanied by impaired mitochondrial function. Instead, it represents a context-dependent adaptive state established through distinct cellular responses to persistent DAMP input and coordinated regulation of the mTOR/HIF-1α/ROS network. From the perspective of trained immunity, these divergent metabolic states may determine whether cells retain altered responsiveness after withdrawal of the initial stimulus and subsequent restimulation or instead shift toward dysfunctional or protective responses. This cell-type specificity is essential for both evidence interpretation and therapeutic design, because metabolic alterations in OA chondrocytes cannot be directly extrapolated to trained immunity in myeloid cells.

Taken together, the evidence supporting the proposed DAMP–trained-immunity framework is derived from substantially different experimental systems and should not be assigned equivalent weight. Some relationships have been directly demonstrated in circulating monocytes from patients with knee OA, whereas others are supported by OA animal or in vitro studies, non-OA trained-immunity models, or mechanistic inference. To clarify these distinctions, the principal mechanistic links discussed above are classified according to their cellular source, OA specificity, experimental design, and fulfillment of the defining criteria for trained immunity (Table 3).

## 7. Potential Links Between Trained Immunity, Inflammatory Memory, and Chronic OA Progression

The pathological significance of trained immunity extends beyond the resetting of cellular states; more importantly, it lies in how this altered state is translated into persistent inflammatory outputs upon subsequent stimulation. In this review, inflammatory memory refers to a broader tissue-level propensity of the joint microenvironment to reactivate inflammatory circuits. In OA, this state may involve trained myeloid cells together with recurrent DAMP release, stromal-cell alterations, defective resolution, crystal deposition, and continuing mechanical injury. Within this broader context, trained innate immune cells may shift from transient activation toward a low-threshold, hyperresponsive reactivation state, thereby promoting inflammatory amplification, cumulative tissue damage, and disruption of joint microenvironmental homeostasis.

### 7.1. Low-Threshold Reactivation and Inflammatory Amplification

The contribution of trained immunity to inflammatory memory is characterized by enhanced functional responses of innate immune cells upon secondary stimulation after an initial activation and recovery phase. This process does not require the secondary stimulus to be identical to the original trigger but instead reflects a generalized tendency toward reactivation determined by persistent alterations in cellular states. In vitro studies have shown that primary human monocytes preconditioned with β-glucan, BCG, or oxLDL exhibit enhanced production of TNF and IL-6 even when exposed to heterologous secondary stimuli, indicating that trained cells acquire a nonspecific capacity for amplified immune responses [95,96]. Cytokine-mediated signaling can also contribute to trained immune programs independently of direct DAMP or PAMP sensing. For example, transient IL-1β exposure can induce persistent functional alterations in human hematopoietic progenitor cells, with their monocyte progeny producing more TNF and IL-1β upon subsequent stimulation [97]. IL-1β should therefore be regarded as an inflammatory cytokine that propagates training-related responses rather than as a DAMP. These findings suggest that persistent inflammation in OA is unlikely to depend solely on the prolonged survival of the same circulating monocytes. Instead, it may be sustained by repeated recruitment of trained monocytes, persistence or local renewal of selected tissue-resident macrophage populations, and continuous generation of hyperresponsive myeloid cells through HSPC reprogramming. Within the OA joint microenvironment, this low-threshold reactivation state can further evolve into sustained inflammatory amplification. FLSs exhibit prominent priming characteristics in response to repeated inflammatory stimulation, and exosomes derived from inflammatory FLS can further enhance macrophage glycolysis and pro-inflammatory functional reprogramming [98,99]. Meanwhile, exosome-like vesicles released from OA cartilage can be taken up by synovial macrophages and promote mature IL-1β production, thereby further amplifying local inflammation [100]. Therefore, in OA, subsequent activation does not necessarily require a new severe injury event. Mild mechanical stress, local metabolic fluctuations, or recurrent exposure to low levels of inflammatory mediators may be sufficient to trigger disproportionate inflammatory outputs from trained immune cells.

### 7.2. Synovitis, Cartilage Degradation, and Dysregulated Subchondral Bone Remodeling

Once the hyperresponsive state established by trained immunity is formed, its consequences extend beyond transient increases in inflammatory mediators and gradually manifest as persistent synovitis, aggravated cartilage degradation, and disrupted subchondral bone remodeling. Among these pathological changes, synovitis represents one of the most prominent tissue manifestations in which inflammatory memory can be amplified. Under persistent stimulation, macrophages and FLS within pathological synovium can establish stable inflammatory amplification loops, maintaining elevated levels of IL-1β, TNF-α, and IL-6 in the joint cavity above baseline levels [99,100]. Among these cytokines, IL-1β and TNF-α primarily contribute to inflammatory initiation and amplification, whereas IL-6 is more closely involved in sustaining local inflammatory circuits. Studies have shown that synovial macrophages and their secreted factors can further induce FLS and chondrocytes to increase the expression of MMP-1, MMP-3, and other matrix-degrading enzymes. Moreover, under obesity-associated conditions, interactions between chondrocytes and FLS further enhance IL-6 production, suggesting that inflammatory networks and metabolic factors exert synergistic amplification effects in OA [101,102,103].

The inflammatory synovial environment subsequently promotes cartilage destruction. Depletion of synovial macrophages markedly reduces aggrecanases, MMPs, and various inflammatory and destructive responses, indicating that macrophages not only participate in maintaining inflammation but also function upstream of cartilage matrix degradation [101]. Therefore, OA cartilage is exposed not merely to transient inflammatory insults but to a continuously remodeled catabolic environment, ultimately resulting in disruption of collagen networks, loss of ACAN, and impaired mechanical properties. In this context, the consequence of trained immunity in cartilage is not limited to the elevation of a specific class of degradative enzymes; rather, it facilitates repeated activation and maintenance of broader catabolic programs.

In addition, dysregulated subchondral bone remodeling represents an extension of inflammatory memory into deeper joint compartments. During early OA development, subchondral bone does not simply undergo sclerosis but experiences dynamic changes involving bone resorption, abnormal bone formation, and vascular and sensory nerve invasion. Studies have demonstrated that leukemia inhibitory factor (LIF) released by osteoclasts promotes abnormal subchondral bone remodeling and accelerates OA progression. Meanwhile, osteoclasts within subchondral bone can induce aberrant sensory nerve growth and vascular invasion, thereby directly linking structural alterations to persistent pain [104,105]. These data support the view that the consequences of trained immunity are not restricted to intra-articular inflammation but may also extend to subchondral bone remodeling through sustained exposure to inflammatory mediators and metabolic signals.

### 7.3. Inflammatory Memory and the Chronic Progressive Phenotype of OA

The clinical significance of inflammatory memory in OA extends beyond simply explaining the persistence of inflammation. More importantly, it suggests that joint tissues may remain in a state of heightened susceptibility to reactivation over prolonged periods. Even when symptoms or inflammatory manifestations temporarily decline, local danger-signal sensing and amplification systems may not fully return to baseline. Therefore, partial remission may represent a transient reduction in inflammatory intensity rather than a true restoration of joint microenvironmental homeostasis. From clinical and imaging perspectives, fluctuations in OA-associated inflammation and symptoms are not entirely stochastic. Studies have shown that greater effusion-synovitis severity is associated with an increased risk of subsequent cartilage damage progression, while synovitis itself exhibits dynamic changes throughout disease progression and correlates closely with symptom variation [106,107]. Longitudinal magnetic resonance imaging (MRI) studies in patients with painful OA have further demonstrated a temporal association between changes in synovitis and fluctuations in pain, supporting the concept that local inflammatory states can undergo periodic reactivation [108]. In addition, OA synovial fluid itself can sustain a pro-inflammatory environment that promotes macrophage polarization, while synovial tissues may undergo renewed thickening and inflammatory cell accumulation during pain recurrence. These observations demonstrate dynamic inflammatory activity but do not, by themselves, establish cellular memory, because recurrent synovitis or pain may also result from continued mechanical injury, altered loading, crystal activation, metabolic fluctuations, or renewed DAMP release [99,109]. Therefore, inflammatory memory in OA may be considered a persistent state of tissue susceptibility. In this state, the joint remains under low-level activation at baseline and shifts rapidly toward amplified inflammation after minor challenges, with trained immunity potentially contributing to the heightened responsiveness. For patients, this state may manifest as recurrent symptoms, shortened remission periods, and fluctuating pain severity. At the disease level, each episode of reactivation may contribute to additional structural damage accumulation, gradually driving OA from a low-grade inflammatory condition toward irreversible chronic progression. This interpretation helps explain why inflammatory symptoms can fluctuate while the underlying joint environment remains vulnerable to reactivation.

## 8. Therapeutic Opportunities Related to DAMP Signaling and Trained Immunity in OA: Current Evidence and Translational Perspectives

The proposed links among DAMP signaling, myeloid-cell functional alterations, and trained immunity suggest several potential intervention opportunities upstream of terminal inflammatory responses. Compared with conventional strategies primarily focused on analgesia and inflammation suppression, interventions potentially relevant to trained immunity can be considered at several levels, including danger-signal input, PRR-mediated sensing, epigenetic and metabolic regulation, and modulation of macrophage functional states. The ultimate goal is to reduce the likelihood that the joint microenvironment repeatedly enters a low-threshold, hyperresponsive state, thereby suggesting possible strategies for delaying chronic OA progression (Figure 3). These approaches should not yet be interpreted as clinically validated trained-immunity therapies for OA. Most available interventions act on related inflammatory, metabolic, or macrophage-regulatory pathways; whether they truly reset trained immune states in OA remains to be tested. Accordingly, the interventions discussed below are classified as demonstrated modifiers of trained immunity, mechanistically plausible candidate modifiers, or conventional OA anti-inflammatory/tissue-protective approaches with potential relevance to training-related pathways.

Representative DAMP- and PRR-directed modulators, together with other approaches potentially relevant to trained immunity, are summarized according to their experimental context, OA-specific evidence, evidence for trained-immunity modification, and principal translational limitations (Table 4).

### 8.1. Targeting Upstream Danger Signals: Blocking DAMP Generation and Enhancing Clearance

Because DAMPs provide upstream inflammatory inputs that may contribute to myeloid-cell training, reducing their persistent release or enhancing their clearance represents a potential strategy for limiting repeated activation and training-related responses within the joint. Unlike approaches targeting terminal inflammatory mediators, this strategy focuses on reducing the endogenous ligand burden that continuously drives PRR activation, thereby weakening the establishment and maintenance of trained states at the source. Among potential upstream targets, HMGB1 has received considerable attention. As a representative alarmin, HMGB1 can be continuously released during chondrocyte injury, matrix degradation, and synovial inflammation. Experimental studies have shown that pharmacological modulation of the HMGB1/TLR4/NF-κB signaling axis can suppress IL-1β-induced inflammatory responses, apoptosis, and matrix degradation-related gene expression in chondrocytes [110]. In addition, glycyrrhizin, an HMGB1 antagonist, has been reported to alleviate OA progression in animal models by inhibiting the HMGB1-RAGE/TLR4-NF-κB/AKT pathway [111]. These findings support HMGB1-related signaling as an upstream therapeutic target in OA. Recent evidence further indicates that HMGB1 neutralization can attenuate synovial inflammation associated with trained circulating monocytes [10]. However, whether HMGB1 blockade prevents the establishment of training or reverses an already established trained state remains unresolved.

Another potential strategy involves reducing sustained DAMP generation. In OA, DAMPs are extensively derived from ECM degradation, cell death, and mitochondrial injury; therefore, protecting chondrocytes from damage may indirectly reduce the burden of danger signals. Previous studies have shown that LDHA inhibition decreases ROS production in chondrocytes and alleviates degenerative changes, suggesting that controlling metabolic injury may reduce the generation of new danger signals [92]. Meanwhile, restoring macrophage-mediated clearance of apoptotic cells through efferocytosis may provide a complementary approach. Hyperglycemic and lactate-rich environments impair efferocytosis and exacerbate synovitis, indicating that enhancing danger signal clearance may represent another means of reducing training inputs [90]. Overall, the significance of blocking DAMP generation or promoting DAMP clearance does not lie in completely eliminating all danger signals, but rather in reducing the probability that the local immune system remains in a continuously trainable and readily reactivatable state. This DAMP-lowering strategy may warrant further evaluation in OA subgroups characterized by high synovial inflammatory burden or prominent metabolic abnormalities.

### 8.2. Modulating PRR-Mediated Signaling Networks and Downstream Inflammatory Amplification Cascades

While controlling DAMP input primarily targets the source of danger signals, inhibiting PRRs and their downstream pathways focuses on regulating danger signal recognition and amplification. The central objective of this strategy is not to completely eliminate DAMPs, but rather to reduce the efficiency with which local cells convert danger signals into inflammatory outputs. Under conditions of persistent low-level DAMP exposure, OA progression is not determined solely by the abundance of individual ligands; instead, it is shaped by an integrated signaling amplification network involving receptors, adaptor proteins, and downstream transcriptional programs. TLR4 represents a representative therapeutic target within this regulatory framework. The TLR4 inhibitor TAK-242 has been shown to markedly suppress pro-inflammatory cytokine production and reduce inflammatory responses in OA-derived FLS, suggesting that TLR4 blockade can directly attenuate inflammatory outputs from local effector cells in OA [112].

In parallel, the NLRP3 inflammasome integrates multiple danger signals, including crystal deposition, mitochondrial injury, and ion homeostasis disruption, ultimately promoting IL-1β maturation. Therefore, targeting NLRP3 may be particularly relevant for OA subgroups with elevated inflammatory burdens [113]. The pharmacological NLRP3 inhibitor CY-09 has been reported to alleviate inflammatory injury in OA chondrocytes. These data support the view that even when DAMP input cannot be completely eliminated, restricting the intracellular integration of danger signals into IL-1β maturation and inflammatory amplification may still reduce the pathological outputs associated with trained immune states [114]. Compared with upstream DAMP reduction strategies, interventions targeting PRRs and their downstream signaling pathways can be viewed as approaches aimed at increasing the activation threshold of local immune systems. For OA subgroups characterized by high inflammatory burden, pronounced synovial responses, or accompanying metabolic disturbances, targeting signaling nodes such as TLR4 and NLRP3 may provide greater immunological and translational potential. For trained immunity, the key question is whether these inhibitors merely suppress acute inflammatory output or also raise the long-term activation threshold of myeloid cells.

### 8.3. Targeting Epigenetic and Metabolic Pathways Associated with Trained Immunity

Epigenetic and metabolic interventions may target molecular pathways involved in the establishment or maintenance of trained states. The underlying rationale is that once trained immunity has been stably established through chromatin remodeling, histone modifications, and metabolic reprogramming, simply suppressing terminal inflammatory outputs may be insufficient to reverse the hyperresponsive state. Instead, interventions targeting the molecular processes that maintain this state may be required. At the epigenetic level, regulation of chromatin-modifying molecules has emerged as a potential therapeutic approach. Studies in OA models have shown that the natural triterpenoid compound obacunone functions as a histone deacetylase (HDAC) inhibitor, reducing p38 mitogen-activated protein kinase (p38 MAPK) activation and alleviating IL-1β-induced chondrocyte injury. In vivo, obacunone treatment also attenuates cartilage degeneration and synovitis [115]. Another study demonstrated that the HDAC inhibitor panobinostat targeting Forkhead box O (FoxO) transcription factors can restore chondrocyte homeostatic programs and reduce cartilage damage in multiple OA models [116]. In addition, bromodomain-containing protein 4 (BRD4) is upregulated in OA cartilage, and pharmacological inhibition of BRD4 with JQ1 suppresses HMGB1/NF-κB signaling and alleviates cartilage inflammation and structural damage in post-traumatic OA. These data support the view that, beyond enzymes responsible for histone modification, regulatory proteins recognizing acetylated histone marks may also serve as potential therapeutic targets in OA [117,118].

On the other hand, restoring metabolic homeostasis may weaken the conditions required for maintaining inflammatory memory. Metformin has been shown to act on multiple cell types, including chondrocytes, synovial macrophages, and adipose tissue, and attenuate OA progression under both high-fat diet and normal diet conditions. Its effects are accompanied by reduced cartilage catabolism, decreased infiltration of pro-inflammatory synovial macrophages, and diminished adipose tissue inflammation [119]. Moreover, metformin alleviates chondrocyte senescence and matrix degradation through the AMPK/mTOR axis, indicating that metabolic interventions may not only improve systemic metabolic conditions but also directly influence the functional states of local immune and tissue cells [120]. Therefore, the significance of these interventions extends beyond suppressing terminal inflammatory effects. Their potential value lies in modulating epigenetic and metabolic processes associated with trained-state maintenance, although whether these interventions reverse trained immunity in OA remains to be determined.

### 8.4. Precision Modulation of Macrophage Functional States Through Targeted Delivery Approaches

Single-target interventions may be insufficient to effectively reset inflammatory memory states in OA. Therefore, redirecting local macrophage functional states in combination with precision delivery systems represents an important complementary strategy. The goal of this approach is to reduce macrophage-mediated amplification of danger signals and promote context-appropriate functional states that support inflammatory resolution and tissue repair. Extracellular vesicles, hydrogels, and biomimetic nanotherapeutic systems provide powerful platforms for achieving this goal through immunomodulation, localized sustained release, and targeted enrichment.

MSC-derived exosomes and microvesicles can alleviate cartilage and bone degeneration, suggesting potential cross-tissue regulatory effects [121]. Chondrocyte-derived exosomes delivered in thermosensitive hydrogels can achieve sustained intra-articular release and promote reparative macrophage states in OA models [122]. Moreover, exosomes derived from macrophages classified as M2-like in the original study alleviated synovitis and cartilage damage through phosphoinositide 3-kinase (PI3K)/AKT/mTOR-associated signaling [123]. Platelet-derived exosomes can reprogram macrophage functional states in rheumatoid arthritis models [124]; however, this represents cross-disease evidence and has not been shown to modify trained immunity in OA. Biomimetic systems, including activated-macrophage-membrane-coated nanoparticles, may further improve the local delivery of immunoregulatory, metabolic, or nucleic-acid therapeutics [125]. Whether these approaches reduce recurrent inflammatory activation or long-term inflammatory memory in OA remains unknown. Near-term studies should prioritize blood- and synovial-fluid biomarkers of trained monocyte states and evaluate their longitudinal associations with synovitis recurrence, structural progression, and treatment response.

## 9. Current Limitations

Although direct evidence has emerged for HMGB1-associated training in circulating monocytes, the broader framework still relies substantially on indirect or extrapolated findings and requires validation across OA populations, disease stages, phenotypes, myeloid-cell compartments, and DAMP classes. Longitudinal studies are also needed to define the temporal relationships among DAMP exposure, trained immune phenotypes, tissue-level inflammatory memory, and OA progression. Second, the relative contributions of different myeloid-cell compartments remain unclear. Circulating monocytes currently have the strongest direct OA-specific support, whereas persistent training of resident or recruited synovial macrophages and hematopoietic progenitor cells has not been directly demonstrated in OA. Third, existing experimental models do not fully recapitulate the complexity of human OA progression. Most trained-immunity studies rely on short-term in vitro stimulation models or acute inflammatory animal models, which may not accurately represent the chronic process of sustained danger-signal exposure, trained-state maintenance, and cumulative tissue damage occurring in OA. Finally, patient heterogeneity has not been sufficiently incorporated into this framework. OA patients differ substantially in inflammatory burden, metabolic status, synovial responsiveness, pain sensitivity, and structural progression rates. Of note, trained immunity is unlikely to dominate all OA phenotypes and may instead represent a permissive immunological state that promotes persistent inflammation in susceptible OA endotypes. Current evidence remains insufficient to identify these patient subgroups or to establish trained immunity as a general mechanism of OA. Therefore, further studies integrating temporal and spatial immune profiling with patient-specific disease stratification are required.

## 10. Conclusions

Overall, accumulating evidence supports potential molecular links among persistent DAMP exposure, PRR-dependent signaling, functional alterations in monocyte–macrophage populations, epigenetic and metabolic reprogramming, and trained immunity in OA. This framework provides an integrated perspective for examining how these processes may contribute to persistent inflammation, progressive tissue damage, and disease evolution. Of note, this concept does not replace established pathogenic mechanisms involving mechanical loading, aging, metabolic abnormalities, and tissue degeneration. Rather, it complements these perspectives by considering adaptive remodeling of innate immunity alongside inflammatory amplification, persistent synovitis, cartilage degradation, and pathological subchondral bone remodeling. Within this framework, injury-associated changes in immune responsiveness represent one potential contributor to inflammatory persistence rather than an established unifying mechanism of OA. Initial direct evidence now supports trained immune reprogramming in knee OA, strengthening the biological basis of this framework. However, its applicability across OA phenotypes, joint compartments, DAMP classes, and disease stages remains unresolved and requires further validation.

Future studies should focus on strengthening causal validation and accelerating clinical translation of this model. First, the emerging direct evidence should be independently replicated to determine whether DAMP-induced trained states are reproducible across OA populations and whether similar responses can be induced by DAMPs other than HMGB1. Further studies should also clarify the relative contributions of circulating monocytes, recruited monocyte-derived macrophages, resident synovial macrophages, and bone marrow HSPCs to inflammatory memory. Second, future investigations should establish more direct mechanistic links between trained immune states and tissue-level pathological outcomes. Specifically, it remains critical to determine whether and how heightened responsiveness of monocyte–macrophage populations contributes to recurrent synovitis, ECM degradation, and dysregulated subchondral bone remodeling. Third, integration of single-cell sequencing, multi-omics approaches, spatial omics, and clinical stratification analyses will be essential for identifying OA subgroups characterized by high inflammatory burden and hyperresponsive immune states. Based on such stratification, combined therapeutic strategies targeting DAMP reduction, PRR desensitization, epigenetic and metabolic reprogramming, and macrophage functional-state modulation may enable the transition from mechanistic hypotheses toward precision disease modification and individualized intervention.

## Figures and Tables

**Figure 1:**
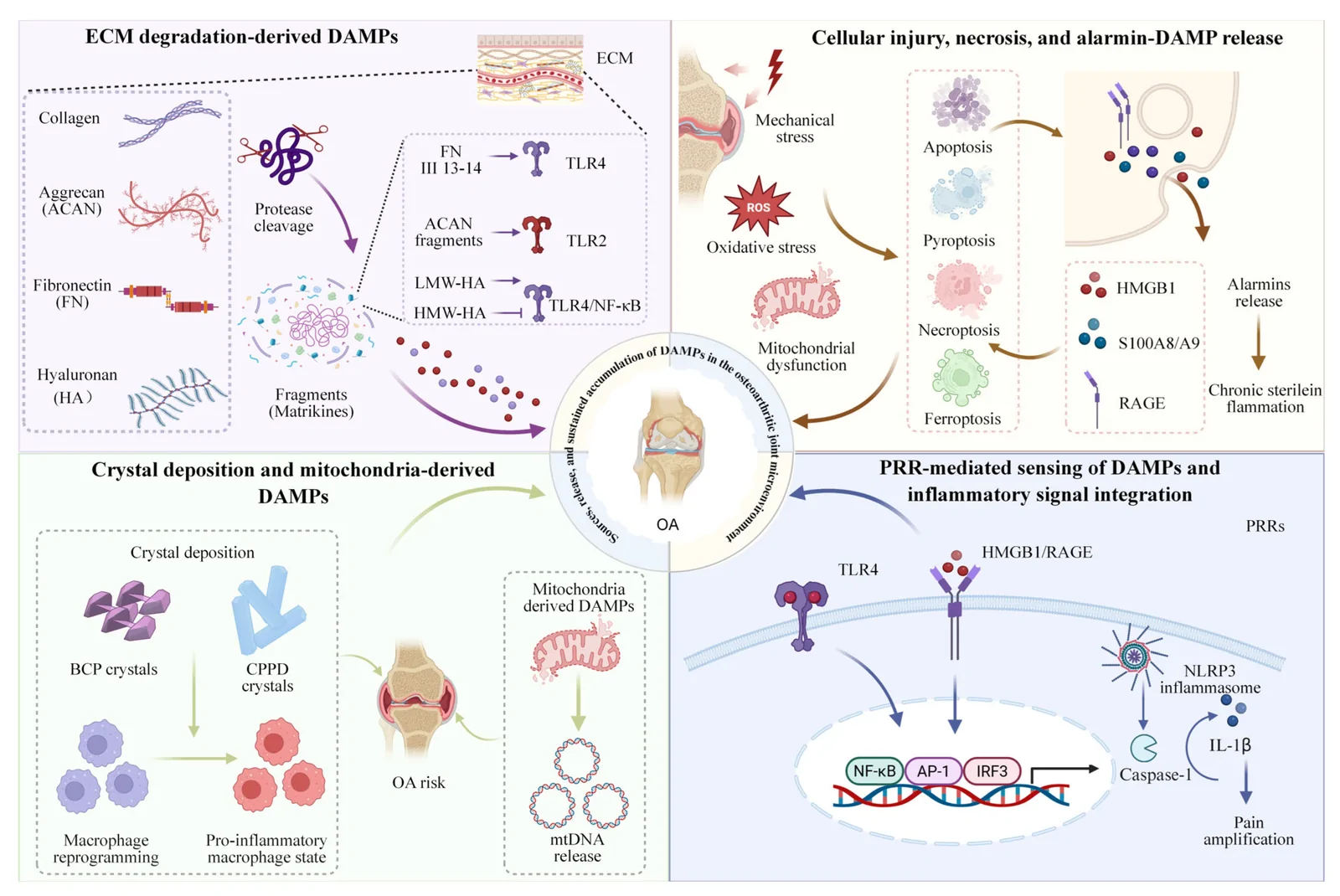
Sources and sensing pathways of DAMPs in the OA joint microenvironment. The four panels illustrate extracellular matrix degradation-derived DAMPs, cellular injury and alarmin release, crystal deposition and mitochondria-derived DAMPs, and the integration of danger-sensing and inflammatory pathways. TLRs and RAGE participate in cell-surface or endosomal danger-signal recognition, whereas NLRP3 functions downstream as a cytosolic inflammasome sensor and signaling platform responding to cellular disturbances rather than as a classical receptor that directly binds a specific extracellular DAMP. The pathways shown summarize DAMP generation, sensing, and inflammatory activation and should not be interpreted as evidence that all displayed DAMPs induce trained immunity.

**Figure 2:**
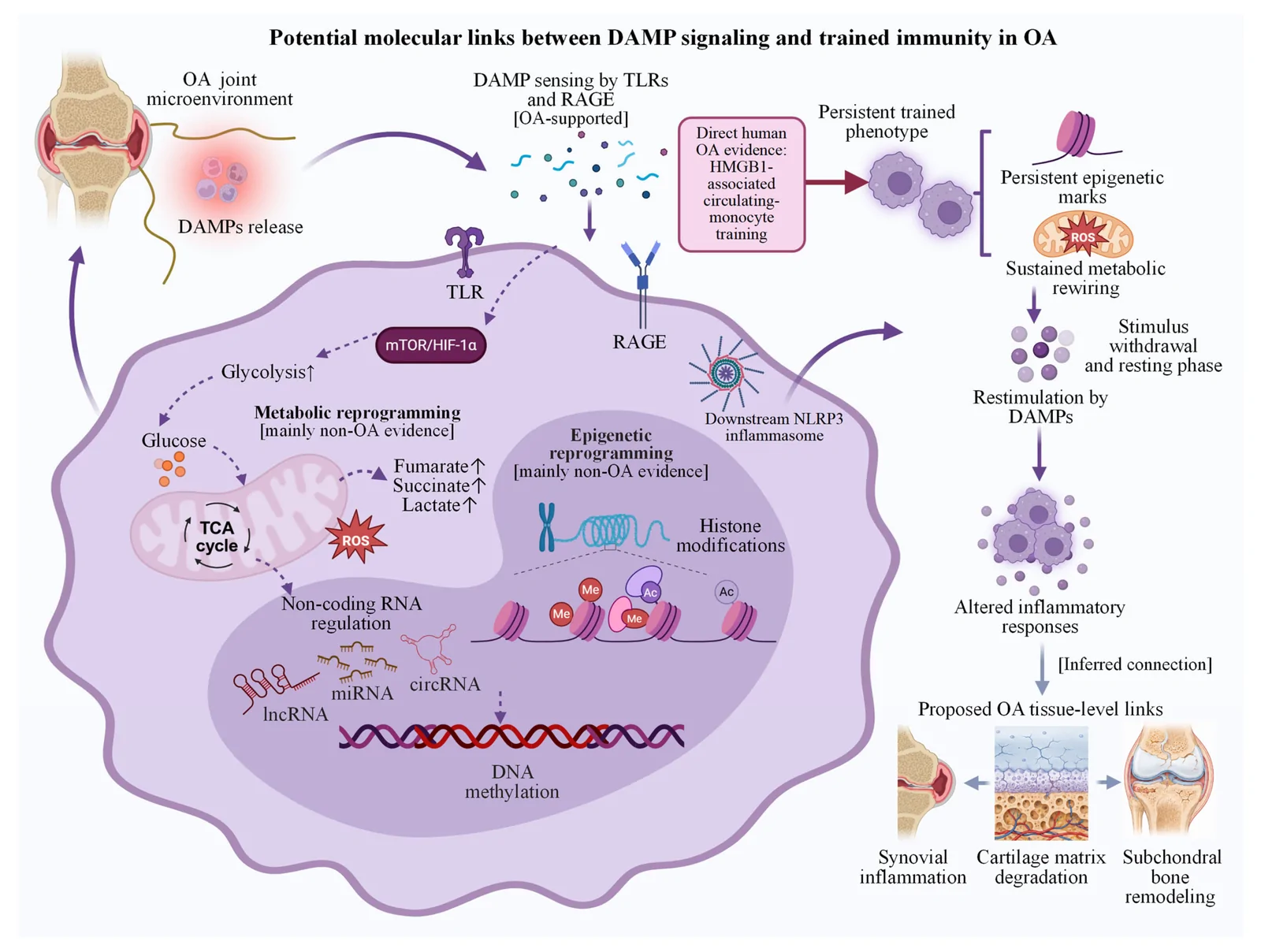
Potential molecular links between DAMP signaling and trained immunity of monocyte–macrophage populations in OA. DAMPs released in the OA joint microenvironment are sensed by cell-surface or endosomal PRRs, including TLRs and RAGE, whereas downstream cellular disturbances may activate the cytosolic NLRP3 inflammasome. Solid purple lines indicate pathways supported by OA-related evidence, dashed purple lines indicate trained-immunity mechanisms established mainly outside OA, and thin gray lines indicate proposed or inferred connections. The burgundy box and solid burgundy arrow highlight HMGB1-associated training of circulating monocytes as the currently established direct human OA example. Other links among PRR signaling, metabolic and epigenetic reprogramming, persistent myeloid responsiveness, and joint-tissue outcomes remain incompletely validated. Following stimulus withdrawal and a resting phase, trained cells may mount altered responses to subsequent challenges, potentially contributing to synovial inflammation, cartilage matrix degradation, and subchondral bone remodeling.

**Figure 3:**
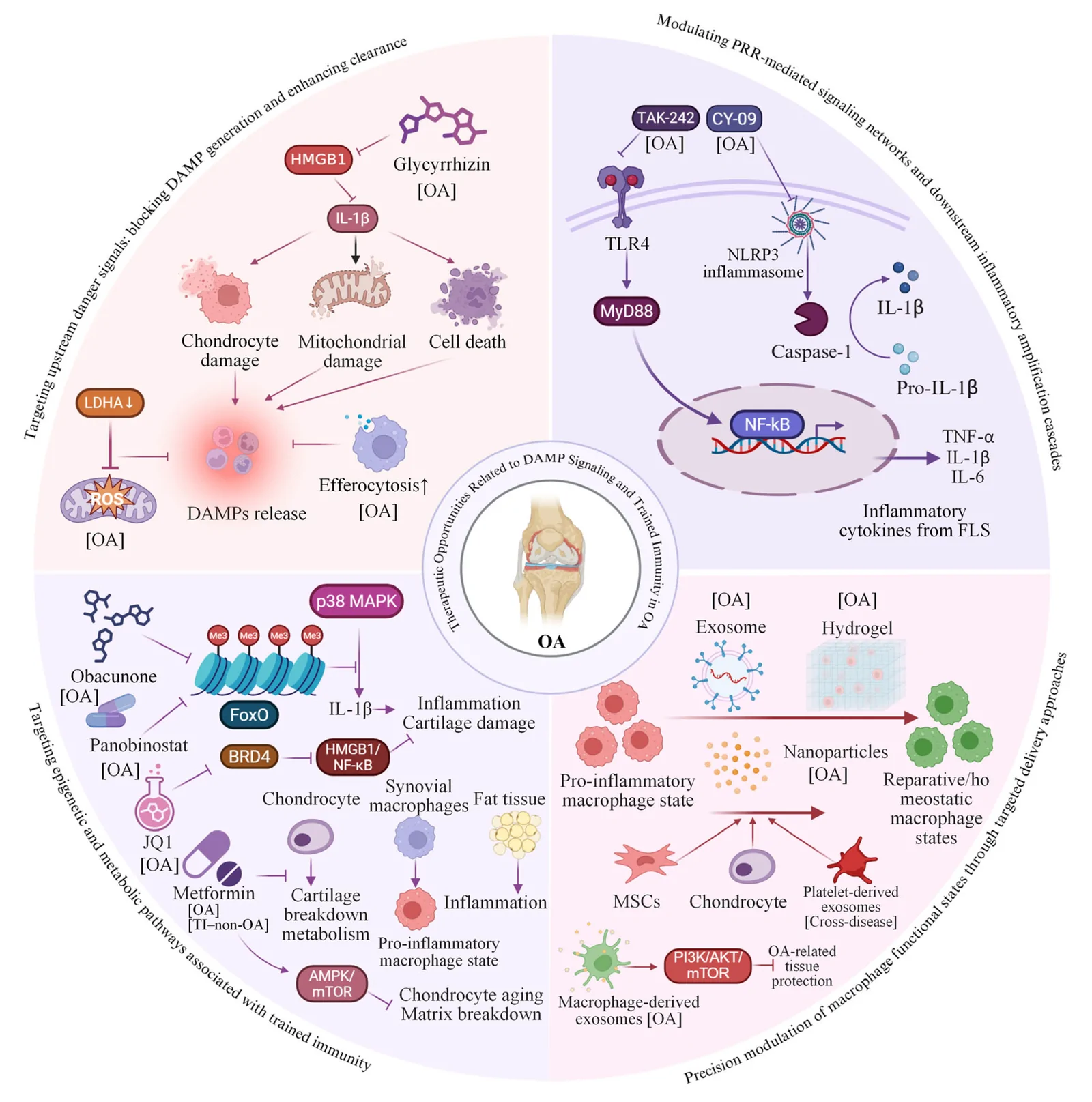
Therapeutic opportunities related to DAMP signaling and trained immunity in OA. The figure summarizes interventions targeting DAMP generation or clearance, PRR-mediated signaling, epigenetic and metabolic pathways, and macrophage functional states. Labels indicate whether an intervention has been evaluated in OA models or samples, has modified trained immunity outside OA, or is extrapolated from another inflammatory disease. Most interventions shown suppress acute inflammation, metabolic dysfunction, macrophage polarization, or tissue damage; these effects do not establish prevention or reversal of trained immunity. No intervention has yet been shown to reverse an established trained state in OA. Arrows indicate promotion or the direction of action, whereas T-shaped lines indicate inhibition.

**Table 1:** Cellular context and evidence level of representative DAMP–PRR relationships in OA.

DAMP	Principal PRR or Sensor	OA-Related Cell or Compartment	Experimental Model	Main Observation	Evidence for Trained Immunity
Fibronectin fragments	TLR2/TLR4	Articular chondrocytes and joint tissues	Human cells and experimental models	MyD88-dependent MMP expression and catabolic signaling	Activation only
HMGB1	TLR2, TLR4/MD-2 and RAGE	Circulating monocytes, chondrocytes and synovial cells	Human knee OA with complementary cell and mouse experiments	Inflammatory signaling and persistent monocyte hyperresponsiveness	Direct evidence in circulating monocytes
Lumican	TLR4	Cartilage, chondrocytes and macrophages	Human OA tissues and experimental studies	Cartilage catabolism and macrophage inflammatory polarization	Not established
Damage-released dsRNA	TLR3	Articular chondrocytes	Cell and animal experiments	IL-33-associated cartilage degeneration	Not established
BCP crystals	NLRP3-related sensing	Monocyte-derived macrophages	Cell and OA-related experimental studies	Inflammatory and metabolic remodeling	Not established
Extracellular mtDNA	Receptor-level OA evidence remains incomplete	Synovial fluid and injured joint tissues	Naturally occurring joint injury	Associated with cartilage damage severity	Not established

Notes: The listed receptors indicate the principal recognition or sensing pathways reported in the cited studies. NLRP3 functions mainly as a cytosolic inflammasome sensor downstream of cellular disturbances and should not be interpreted as a classical receptor that directly binds every extracellular DAMP. Evidence of acute PRR activation, inflammatory mediator production, or macrophage polarization does not by itself demonstrate trained immunity. Direct OA-specific evidence for a DAMP-induced trained phenotype is currently strongest for HMGB1 in circulating monocytes.

**Table 2:** Operational criteria for distinguishing trained immunity from persistent activation and transient priming.

Criterion	Required Interpretation
Initial stimulation	Cells receive a defined primary microbial, endogenous, metabolic, or inflammatory stimulus
Stimulus withdrawal	The primary stimulus is removed by washing or withdrawal
Resting phase	Cells are maintained for a defined interval sufficient to distinguish memory from acute activation
Secondary challenge	A homologous or heterologous challenge is administered
Altered secondary response	Cytokine production, phagocytosis, antimicrobial activity, migration, or another functional output differs from untrained controls
Persistence	The altered response remains detectable beyond the acute effects of the primary stimulus
Molecular support	Persistent chromatin, histone, DNA methylation, transcriptional, or metabolic changes support the functional phenotype
Mechanistic validation	Genetic or pharmacological interruption of the proposed epigenetic or metabolic pathway weakens the trained response

Notes: Persistent activation depends on continued stimulus exposure, whereas priming enhances an immediate response without necessarily producing durable memory. Endotoxin tolerance is characterized by reduced responsiveness to repeated stimulation, while trained immunity is identified by a persistent increase or qualitative alteration in the response after stimulus withdrawal and restimulation. Macrophage polarization describes a context-dependent functional state and does not, by itself, establish trained immunity.

**Table 3:** Evidence hierarchy for the proposed DAMP–trained-immunity framework in osteoarthritis.

Proposed Mechanistic Link	Experimental System and Cell Type	Principal Finding	Training–Rest–Restimulation Design	Direct Evidence of Trained Immunity	Source-Based Evidence Category	References
HMGB1-induced trained state in circulating monocytes	Human knee OA circulating monocytes; complementary mouse and cell experiments	HMGB1 promoted a persistent hyperresponsive monocyte state through MyD88–IRF1 signaling, TRIM28-mediated SUMOylation, and CXCR4-dependent synovial migration.	Partially addressed: 2-day ex vivo rest followed by HMGB1 restimulation; antecedent in vivo training exposure was not experimentally timed	Yes, with design limitations	A	[10]
Systemic priming of circulating monocytes in OA	Peripheral blood and synovial-fluid monocytes from patients with OA	OA monocytes display increased activation markers and inflammatory cytokine production; CD14-positive monocytes and soluble CD14 are associated with progression.	No	No	A	[50,51]
Chemokine-dependent recruitment of inflammatory monocytes	Human knee OA samples and experimental OA models	The CCL2–CCR2 and CCL3–CCR1 axes promote monocyte recruitment, synovitis, and cartilage damage.	No	No	B	[48,49]
Resident and recruited macrophage heterogeneity	Human/animal synovium and OA synovial fluid	Homeostatic resident macrophages and inflammatory recruited monocyte-derived macrophages occupy distinct states and participate differently in barrier function and inflammatory amplification.	No	No	B	[52,53,54,55,56,57]
DAMP–pattern-recognition-receptor signaling in joint cells	OA chondrocytes, macrophages, cartilage explants, and animal models	Extracellular-matrix fragments, HMGB1, lumican, and damaged-cell RNA activate TLR2, TLR3, TLR4, RAGE, and MyD88-dependent inflammatory pathways.	No	No	B	[11,12,13,14,15,16,60,61,62,63,64,65,66]
Crystal-driven myeloid inflammatory and metabolic remodeling	Basic calcium phosphate crystals; monocyte-derived macrophages and OA-related models	Particulate DAMPs promote inflammatory polarization and alter macrophage metabolic state.	No	No	B	[22,23]
OA-associated chromatin and epigenetic remodeling	Human OA cartilage/chondrocytes and peripheral-blood epigenomic studies	OA tissues show altered chromatin accessibility, enhancer activity, histone regulation, and DNA methylation.	No	No	B	[69,72,73,76,77]
Epigenetic memory in trained monocytes	Human primary monocytes trained with oxidized low-density lipoprotein (oxLDL) or β-glucan	Training produces persistent proinflammatory responsiveness with characteristic chromatin and histone-mark remodeling.	Yes	Yes, outside OA	C	[42,67,70]
Fumarate–KDM5 coupling of metabolism and chromatin	β-glucan-trained human monocytes	Glutaminolysis-derived fumarate inhibits KDM5, supporting maintenance of histone H3 lysine 4 trimethylation and enhanced recall responses.	Yes	Yes, outside OA	C	[71]
Central training of hematopoietic progenitors	Hematopoietic stem and progenitor cells in non-OA inflammatory/training models	Long-lived epigenetic and metabolic programs in progenitors can be transmitted to myeloid progeny.	Model-dependent	Yes, outside OA	C	[43,68]
Glycolytic and mitochondrial rewiring	β-glucan- or oxLDL-trained monocytes/macrophages; OA chondrocytes	Aerobic glycolysis, tricarboxylic-acid-cycle remodeling, and mitochondrial adaptation support trained immunity; OA cells also exhibit major metabolic shifts.	Yes in non-OA training models	Indirect for OA	C/D	[84,85,86,87,91,92,93,94]

Notes: Evidence categories were defined according to the source, disease specificity, and experimental context of the supporting evidence: A, direct evidence obtained from human osteoarthritis (OA) samples; B, OA-specific evidence from animal or in vitro studies supporting an individual component of the proposed framework; C, established trained-immunity evidence derived from non-OA experimental models; and D, mechanistic inference or hypothesis. Evidence category and direct evidence of trained immunity represent two distinct dimensions of evaluation. Category A indicates that the evidence was obtained directly from human OA samples but does not necessarily mean that trained immunity was directly demonstrated. Direct evidence of trained immunity was defined by a primary training stimulus, subsequent stimulus withdrawal or a rest period, restimulation, and persistent functional enhancement, preferably supported by epigenetic and/or metabolic reprogramming. “Yes, outside OA” indicates that these criteria were fulfilled in established trained-immunity models but have not yet been directly validated in OA-derived cells or OA-specific experimental systems. For the HMGB1 study, the resting and restimulation components were functionally assessed, whereas the duration of antecedent in vivo HMGB1 exposure and the restimulation incubation was not explicitly reported; this evidence was therefore classified as direct but not as a fully controlled in vitro training–washout–restimulation experiment.

**Table 4:** Representative DAMP- and PRR-directed modulators and other therapeutic approaches potentially relevant to trained immunity in OA.

DAMP, PRR, or Therapeutic Target	Representative Intervention	Experimental Context	OA-Specific Evidence	Evidence for Trained-Immunity Modification	Main Translational Limitation	References
HMGB1 signaling	HMGB1 neutralization; glycyrrhizin	Human knee OA monocytes; OA chondrocytes and animal models	Yes	Training-associated inflammation was attenuated, but prevention or reversal of established training was not demonstrated	Direct training-related evidence is currently limited to HMGB1 and requires independent validation	[10,110,111]
TLR4 signaling	TAK-242	OA-derived fibroblast-like synoviocytes and experimental OA models	Yes	Not demonstrated	Evidence mainly reflects suppression of acute inflammatory signaling	[112]
NLRP3 inflammasome	CY-09 and related inhibitors	OA chondrocytes and experimental OA models	Yes	Not demonstrated	Effects on trained immunity in OA-derived monocytes or macrophages remain unknown	[113,114]
Epigenetic regulation	Obacunone; panobinostat; JQ1	OA chondrocytes and animal models	Yes	Not demonstrated in OA	Tissue protection or inflammatory suppression does not establish erasure of trained epigenetic memory	[115,116,117,118]
Metabolic regulation	Metformin	Non-OA trained monocytes; OA cells and animal models	Yes	Demonstrated in non-OA trained-monocyte models, but not in OA-derived myeloid cells	Cell-specific effects and reversal of established training in OA remain uncertain	[85,119,120]
Extracellular vesicle and hydrogel delivery	MSC-derived vesicles; chondrocyte-derived exosome hydrogels; macrophage-derived exosomes	OA cell and animal models	Yes	Not demonstrated	Standardization, dosing, durability, and long-term safety require evaluation	[121,122,123]
Platelet-derived exosomes	Platelet-derived exosomes	Rheumatoid arthritis models	No	Not demonstrated	Cross-disease extrapolation; efficacy and immune-memory effects in OA remain unknown	[124]
Biomimetic nanoparticles	Macrophage membrane-coated nanoparticles	Experimental OA models	Yes	Not demonstrated	Targeted delivery does not establish modification of trained immunity	[125]

Notes: “OA-specific evidence” indicates that the intervention was evaluated in human OA samples or experimental OA models. “Modification of trained immunity” requires evidence obtained using a training–rest–restimulation design and does not refer solely to suppression of acute inflammation, macrophage polarization, or tissue damage.

## Data Availability

No new data were created or analyzed in this study. Data sharing is not applicable to this article.

## Abbreviations

The following abbreviations are used in this manuscript:

ACAN	aggrecan
AKT	protein kinase B
AMPK	AMP-activated protein kinase
AP-1	activator protein-1
ATAC-seq	assay for transposase-accessible chromatin using sequencing
ATP	adenosine triphosphate
BCG	Bacillus Calmette–Guérin
BCP	basic calcium phosphate
BMI	body mass index
BRD4	bromodomain-containing protein 4
C/EBPβ	CCAAT/enhancer-binding protein beta
CCL2	C-C motif chemokine ligand 2
CCL3	C-C motif chemokine ligand 3
CCR1	C-C motif chemokine receptor 1
CCR2	C-C motif chemokine receptor 2
CD11b	cluster of differentiation 11b
CD14	cluster of differentiation 14
CD16	cluster of differentiation 16
circRNA	circular RNA
CPPD	calcium pyrophosphate dihydrate
CX3CR1	C-X3-C motif chemokine receptor 1
CXCR4	C-X-C motif chemokine receptor 4
DAMP	damage-associated molecular pattern
DNA	deoxyribonucleic acid
dsRNA	double-stranded RNA
ECM	extracellular matrix
FLS	fibroblast-like synoviocyte
FN	fibronectin
FoxO	forkhead box O
FXR	farnesoid X receptor
G0S2	G0/G1 switch 2
H3K4me3	histone H3 lysine 4 trimethylation
H3K27ac	histone H3 lysine 27 acetylation
H3K36me1/2	histone H3 lysine 36 mono-/dimethylation
HA	hyaluronan
HDAC	histone deacetylase
HIF-1α	hypoxia-inducible factor 1 alpha
HLA-DR	human leukocyte antigen-DR
HMGB1	high-mobility group box 1
HMW-HA	high-molecular-weight hyaluronan
HSPC	hematopoietic stem and progenitor cell
IDH3α	isocitrate dehydrogenase 3 alpha
IL	interleukin
IL1A	interleukin 1 alpha
IL1R1	interleukin 1 receptor type 1
IPL	immune gene-priming long non-coding RNA
IRF1	interferon regulatory factor 1
IRF3	interferon regulatory factor 3
KDM5	lysine demethylase 5
KLF4	Krüppel-like factor 4
LDHA	lactate dehydrogenase A
LIF	leukemia inhibitory factor
LMW-HA	low-molecular-weight hyaluronan
lncRNA	long non-coding RNA
LPS	lipopolysaccharide
LYVE1	lymphatic vessel endothelial hyaluronan receptor 1
MAPK	mitogen-activated protein kinase
MD-2	myeloid differentiation protein 2
MERTK	MER proto-oncogene, tyrosine kinase
MGMT	O6-methylguanine-DNA methyltransferase
MIF	macrophage migration inhibitory factor
miRNA	microRNA
MMP	matrix metalloproteinase
MRI	magnetic resonance imaging
MSC	mesenchymal stem cell
MSU	monosodium urate
mtDNA	mitochondrial DNA
mTOR	mechanistic target of rapamycin
MyD88	myeloid differentiation primary response 88
NF-κB	nuclear factor kappa B
NLRP3	NOD-, LRR- and pyrin domain-containing protein 3
NSD1	nuclear receptor-binding SET domain protein 1
OA	osteoarthritis
OSM	oncostatin M
OSMR	oncostatin M receptor
oxLDL	oxidized low-density lipoprotein
OXPHOS	oxidative phosphorylation
PAMP	pathogen-associated molecular pattern
PI3K	phosphoinositide 3-kinase
PRR	pattern recognition receptor
RAGE	receptor for advanced glycation end products
RNA	ribonucleic acid
ROS	reactive oxygen species
S100A8/A9	S100 calcium-binding proteins A8/A9
sCD14	soluble CD14
Siglec-5	sialic acid-binding immunoglobulin-like lectin 5
SOCS2	suppressor of cytokine signaling 2
SOX9	SRY-box transcription factor 9
SUMO	small ubiquitin-like modifier
TCA	tricarboxylic acid cycle
TLR	Toll-like receptor
TNF	tumor necrosis factor
TREM2	triggering receptor expressed on myeloid cells 2
TRIM28	tripartite motif-containing protein 28
